# Changing the influence of the egocentric reference frame impacts deviations in haptic parallelity matching

**DOI:** 10.1007/s00221-019-05596-x

**Published:** 2019-07-10

**Authors:** Hanneke I. Van Mier

**Affiliations:** Department of Cognitive Neuroscience, Faculty of Psychology and Neuroscience, Maastricht University, P.O. Box 616, 6200 MD Maastricht, The Netherlands

**Keywords:** Haptic perception, Egocentric, Allocentric, Gender, Frame of reference, Parallelity

## Abstract

The large systematic deviations in haptic parallelity matching are most likely due to the biasing influence of the hand-centered egocentric reference frame. Previous results showed that eliminating or reducing this bias resulted in smaller deviations, with significantly larger effects observed in female participants. The current study investigated the effect of reducing the egocentric bias in a pure haptic condition. Blind-folded male and female participants had to feel the orientation of a reference bar with their non-dominant hand and to parallel this orientation on a test bar with their dominant hand. In one condition, they were instructed to use their flat-stretched hand to feel and match the bars, while in the other condition (HPF), they were instructed to set the test bar while gripping the bar with the fingers and thumb. It was hypothesized that the latter would reduce the biasing influence of the hand-centered egocentric reference frame. Results showed that this was indeed the case. Deviations were significantly smaller for HPF; however, this effect was the same in both genders. The previously observed gender effect, showing a significantly larger improvement for women when reducing the influence of the egocentric reference frame, was not replicated.

## Introduction

A spatial orientation task that might seem to be easily and veridically performed often results in large errors when the task has to be performed haptically without visual input. The task at hand involves two bars that need to be paralleled with regard to their orientation. In the so-called haptic parallelity task (Kappers 1999; Kappers and Koenderink 1999), participants have to set two bars that are placed at different positions parallel to each other using only the haptic modality. The orientation of a reference bar is felt with one hand, while the other hand has to rotate a test bar to match the orientation of the latter to the orientation of the former. Performing this task without visual input results in a mismatch between what is perceived as being parallel and what is physically parallel. Participants’ settings often deviate considerably from parallelity (e.g., Fernández-Díaz and Travieso 2011; Kaas and Van Mier 2006; Kappers 1999, 2003; Van Mier 2013, 2016). When participants would perform this task using only an allocentric reference frame, veridical performance would be expected. However, when participants would rely solely on an egocentric reference frame, deviations would be very large. Because the observed deviations in this task have been found to be intermediate between what would be parallel in an allocentric and an egocentric reference frame, Kappers (2002, 2003) suggested that performance on the haptic parallelity task is most likely determined in an intermediate frame of reference in line with performance in reaching and grasping tasks (Flanders and Soechting 1995; Soechting and Flanders 1992). The observed deviations seem to be the result of the use of an allocentric reference frame fixed in external space and an egocentric reference frame which is centered internally. The deviations in orientation perception in the parallelity task are thought to be caused by a biasing influence of the egocentric reference frame (Kappers 2004; Van Mier 2014). The influence of the egocentric reference frame has been found to be present in different settings of haptic matching tasks like in the horizontal plane (e.g., Kaas and Van Mier 2006; Kaas et al. 2007a, b; Kappers 1999, 2018; Newport et al. 2002; Van Mier 2013, 2016; Zuidhoek et al. 2003), the frontoparallel plane (Hermens et al. 2006; Volcic et al. 2007), the midsagittal plane (Kappers 2002, 2004), in three-dimensional space (Volcic and Kappers 2008), behind the back (Fernández-Díaz and Travieso 2011) or behind the head of the participant (Kappers et al. 2018).

While different egocentric reference frames have been suggested for haptic tasks, including a hand- and shoulder-centered (e.g., Luyat et al. 2001), an arm-centered (e.g., Flanders and Soechting 1995; Soechting and Flanders 1992), and a body-centered frame (e.g., Millar and Al-Attar 2004), the nature of the deviations related to haptic parallelity matching indicates a hand-centered reference frame. When participants have to parallel the orientation of a reference bar, which is located at the left side, on a test bar that is positioned at the right side, the right bar has to be rotated in a clockwise direction to be perceived as being parallel to the left bar (e.g., Kaas and Van Mier 2006; Kaas et al. 2007b; Kappers 2004; Van Mier 2013, 2016). Having to parallel the orientation of a reference bar felt at the right side at a test bar located at the left side of the body resulted in rotations in counter-clockwise direction (Kappers 2004, 2018). These systematic deviations in direction suggest that the egocentric reference frame used in the haptic parallelity task is most likely fixed to the hand. It has been found that deviations become larger when the distance between the hands increases horizontally (Fernández-Díaz and Travieso 2011; Kaas and Van Mier 2006; Kappers 1999; Zuidhoek et al. 2003; Van Mier 2013), but not vertically (Fernández-Díaz and Travieso 2011; Kappers and Koenderink 1999). In the former, the orientation of both hands changes when the distance between the hands is changed, while this is not the case with a change in the vertical direction. The size of the deviations has also been found to be participant dependent with large variations between participants (ranging from 8° to 91° reported by Kappers 2003 in a study including 68 participants) and have been replicated by others (e.g., Kappers 2004, 2007, 2018; Kappers and Liefers 2012; Van Mier 2013, 2016; Volcic et al. 2008).

Additional evidence for a hand-centered egocentric reference frame comes from studies involving different hand positions (Kappers and Viergever 2006), different angles between the hands (Kappers and Liefers 2012), and reducing (Van Mier 2013) or eliminating the use of the hand (Van Mier 2016). In the first two studies, the magnitude of the deviations was dependant on the orientation or angle of the hands (Kappers and Liefers 2012; Kappers and Viergever 2006). In the latter two experiments, the bias of the hand-centered egocentric reference frame was reduced or eliminated, because the orientation had to be drawn (Van Mier 2013) or stated verbally (Van Mier 2016). This resulted in a significant decrease in deviations compared to the deviations in the standard haptic parallelity task.

A robust finding is the observation that female participants have significantly larger deviations when haptically paralleling the test bar to the reference bar than male participants (Hermens et al. 2006; Kaas and Van Mier 2006; Kappers 2003, 2007; Zuidhoek et al. 2007; Van Mier 2013, 2016; Volcic et al. 2008). After participants were asked to make 15 bars, placed at various locations, parallel to each other, Kappers (2005) asked the participants to place their hand in a spontaneous and natural way at these same locations. A large correlation between the haptic settings and the hand orientations was found. She found that a weighted average model of allo- and egocentric referencing could best describe the obtained deviations (Kappers 2007). Furthermore, the relative contributions of ego- and allocentric reference frames were such that females showed larger egocentric weighing factors than males. Results from later studies imply that men are most likely better able to overcome the egocentric bias of the hand when haptically matching orientations (Van Mier 2013, 2016; Zuidhoek et al. 2007). Zuidhoek et al. (2007) found no gender differences when participants had to rotate a bar to match a given clock time. When the same participants had to report the orientation of a bar as a clock time, men outperformed women. While both instructions stimulated the use of an allocentric reference frame, as Van Mier (2014) hypothesized, it is likely that in the latter condition, participants feel the orientation of the bar with their flat-stretched hand, while in the former condition, the bar might have been rotated gripping the bar with the fingers. The bias of the hand is more pronounced when using the flat-stretched hand than when using the fingers, which might explain the observed gender difference.

In conditions favouring more allocentric processing, but still requiring the same matching response with the hand, deviations were significantly smaller than in the regular haptic parallelity task. However, these decreases were more or less similar in both genders and still showed a significant gender difference. This was observed when a delay of 10 s was inserted between feeling and matching the orientation (Zuidhoek et al. 2007) and when informative vision was added (van Mier 2013). Those conditions most likely elicit the use of a more allocentric reference frame (Van Mier 2014) resulting in smaller deviations. However, because the involvement of the hand when orienting the reference bar in these conditions is the same as in the regular parallelity task, deviations for female participants were still significantly larger than for male participants due to the fact that men are most likely better at overcoming the egocentric bias of the hand.

As mentioned above, Van Mier (2013, 2016) reduced or eliminated the use of the hand in her studies, where participants had to parallel the orientation of the reference bar by drawing the orientation of test bar (Van Mier 2013) or by verbally naming the code of the line read out on a test protractor that they thought paralleled the orientation of the reference bar (Van Mier 2016). In both studies, significant gender-related differences in deviations were found for the regular condition of the haptic parallelity task with male participants having smaller deviations than female participants. However, deviations between men and women were not significantly different in the conditions in which the orientation had be drawn or named verbally. In these conditions, the biasing effect of the hand-centered egocentric reference frame was reduced or eliminated. Results from other haptic parallelity studies, in which non-significant gender differences have been reported, also indicate that this might be due to a reduction or elimination of this bias. Kappers and Liefers (2012) found no significant gender-related differences in deviations in a haptic parallelity task in which there was no horizontal distance between the hands. In this study, the exploration of the reference bar and matching at the test bar was performed at the same orientation horizontally with both bars being located directly in front of the participant. Both hands performed in a different plane vertically with one hand performing above the other hand. However, caution is needed interpreting the gender differences in this study, because only six participants of each gender were tested. Including a condition, in which the use of the hands was completely eliminated, also resulted in the abolition of gender differences (Kappers and Schakel 2011). In this study, a condition was included in which the participants could see the setup and bars, but matching the orientation of the reference bar at the test bar was done by the experimenter who was instructed by the participant. Recently, Kappers et al. (2018) asked participants to perform the haptic parallelity task behind the head. Despite the large deviations found in this study, deviations of female and male participants were not significantly different. The authors state that this might be due to the small distance between the bars, being 5 and 13 cm. While this is a plausible explanation, the non-significant gender effect might also be due to the position of the hands. To perform the parallelity task behind the head, hands were not aligned in the same parallel direction, but were oriented in a convergent position, rotated towards each other. In addition, because a smaller setup was used with shorter bars, participants used their fingers to feel and match the orientations, as shown in the second figure of their paper. These differences in the position of the hands and the use of the fingers compared to the regular parallelity task might have reduced the egocentric bias of the hands, and subsequently differences between the genders.

The aim of the current study was to determine if deviations would decrease when the egocentric bias of the matching hand was reduced in a different way as studied before. In the study of Van Mier (2013), where participants had to draw the orientation of the reference bar, participants had full view of their drawing hand. This might have additionally stimulated the use of an allocentric reference frame. In the current study, the effect of reducing the egocentric bias of the hand was studied in a pure haptic condition. Deviations in the regular haptic parallelity task, where participants felt and matched the bars using the flat-stretched hand, were compared to deviations in a condition in which the test bar had to be set with the fingers and thumb gripping the bar (see Fig. 2). In the latter condition, the bar was moved by the fingers instead of by the flat-stretched hand. When both bars are felt and manipulated with the flat-stretched hand, participants will try to align both hands resulting in the observed systematic and often large deviations, especially when the distance between the hands is large (up to 120 cm) (Kappers 2003). In this condition test, hand and upper arm will be aligned. When rotating the test bar with the fingers, it was expected that participants would not try to align both hands. In addition, test hand and forearm will not be aligned. In this condition, an arm-centered reference frame might play a role. In addition, participants might use an allocentric cue, such as a clock time. It was hypothesized that matching the test bar while gripping it with the fingers would reduce the bias of the hand-centered egocentric reference frame resulting in smaller deviations. Due to the latter, it was additionally speculated that this effect would be more pronounced in female participants resulting in a larger improvement for female participants than for male participants in that condition.

## Methods

### Participants

Twenty adults (10 men and 10 women), with a mean age of 24 years (SD 1.8, range 20–26 years), took part in the experiment. Mean age of the male participants was 24 years (SD 2.3, range 20–26 years), while the mean age of the female participants was 23.5 years (SD 1.3, range 22–25 years). One participant showed left-handed dominance, the other participants were right-handed as assessed by a Dutch translation of the hand preference questionnaire of Annett (Annett 2004). Participants were all students at Maastricht University, 6 female and 6 male participants were students at the Psychology and Neuroscience Faculty, while the other 8 participants studied at one of the other Maastricht University faculties. Participants were remunerated for their participation and were naïve with respect to the objectives of the study and did not receive feedback on their performance. Informed written consent was obtained prior to the experiment. The study was approved by the local ethics committee and was performed in accordance with the Declaration of Helsinki of 1964.

### Setup and apparatus

**Fig. 1:**
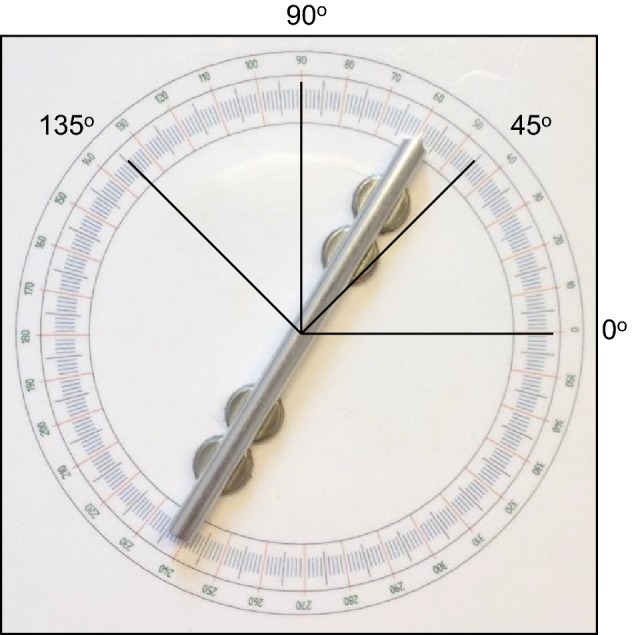
Protractor with the four orientations used in the study. Here, the reference bar is shown with the four magnets

The setup was the same as used in the previous studies (Van Mier 2013, 2016). Two identical iron plates (30 × 30 cm) were used which were covered with a plastic layer on which a protractor with a radius of 10 cm was printed (see Fig. 1). An aluminum bar with a length of 20 cm and a diameter of 1.1 cm was placed on each protractor, of which one was used as reference bar and the other as test bar. Both bars had a small pin attached in the middle which fitted in a hole in the centre of the protractor in such a way that the bars could be rotated 360° and could be positioned accurately in the reference orientations. To increase the resistance against involuntary movement of the bars, small magnets were attached under both bars. Two extra magnets were attached under the reference bar to prevent participants from accidently rotating this bar while exploring its orientation. The bars had an arrow-shaped end on one side, which made it possible to accurately read the orientation of the reference and test bar with a precision of about 0.5°. To avoid moving or shifting of the plates during the experiment, an anti-slip mat was placed under each plate. The plates were positioned at equal distance from the midline of the participant’s body. The distance between the centres of the plates was set at twice the arm length of the participant, which was measured from the shoulder to the wrist. This way each participant was able to comfortably reach both bars.

### Experimental conditions

**Fig. 2:**
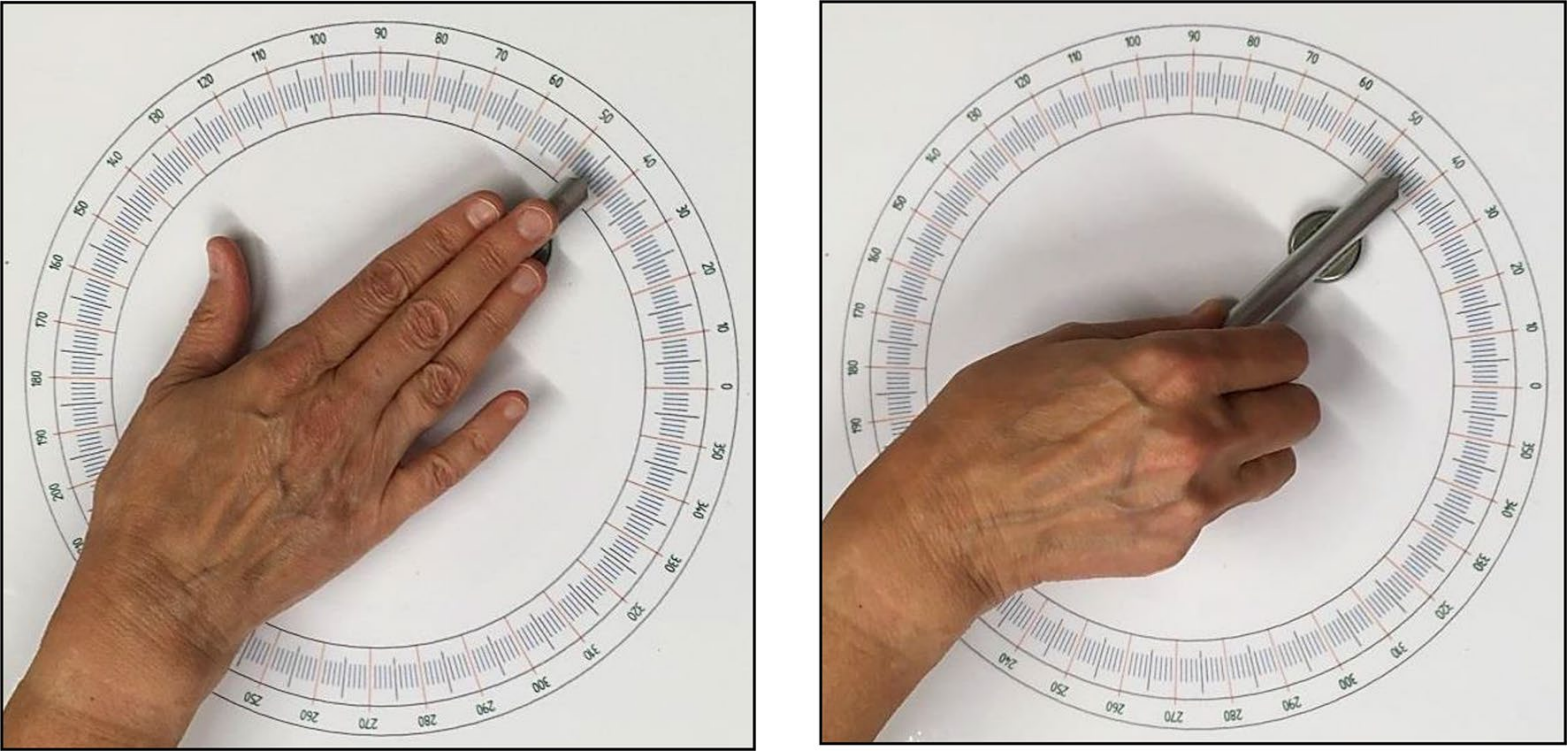
Picture of the hand while setting the test bar. The left picture shows the HPH condition in which the flat-stretched hand was used to rotate the test bar and the right picture shows the HPF condition in which the test bar was rotated using the fingers

Two conditions of the haptic parallelity task were used in the current study. Participants were blindfolded and had to feel the orientation of the reference bar with their non-dominant hand, while they had to parallel this orientation with the dominant hand at the reference bar. Parallel setting was performed bimanually. During the duration of each trial, both bars were touched simultaneously. In both conditions, the orientation of the reference bar had to be explored using the flat-stretched non-dominant hand. Usually, participants perform the haptic parallelity task by feeling and matching the bars using their flat-stretched hands, with the middle finger aligned to the orientation of the bar. This was also the case in the current study. In the occasional instance participants did not stretch the hand, they were instructed by the experimentor to do so. Participants started the study with the condition in which the test bar was haptically paralleled to the reference bar using the flat-stretched hand (HPH) (see Fig. 2, left picture). In the second condition, participants were instructed to orient the test bar while gripping the bar with their fingers and thumb in such a way that the rotating movement was performed by all fingers (HPF) (see Fig. 2, right picture). The order of the two conditions was the same for all participants. They started with the condition in which the test bar had to be oriented using the flat-stretched hand (HPH) followed by the condition in which the test bar was rotated, while the fingers gripped the bar (HPF).

Four different reference orientations were used to study the effect of orientation, 0°, 45°, 90°, and 135°. The 0° orientation was parallel to the horizontal direction and 90° to the vertical direction with 45° and 135° in between (see Fig. 1). Each orientation was repeated three times in each condition. The order of orientation and repetition was presented random within each condition and different for each participant, making sure that the same orientation was never presented consecutively.

### Procedure

Before the experiment started, the arm length of the participant was measured as described above. After participants had signed the informed consent, they filled out the handedness questionnaire to establish if they were left- or right-handed. Next participants received instructions regarding the task and were asked to put two pens in a parallel position using different orientations to make sure that they fully understood the principle of parallelity.

Participants were then blindfolded and the distance between the bars was set at twice the arm length and it was assured that participants were seated in the middle of the two plates. For right-handed participants, the test bar was positioned at the right side of the body, while the test bar was located at the left side of the body for the left-handed participant. Mean arm length of the male participants was 54.0 cm (SD 4.7) and of the female participants 49.3 cm (SD 3.2). Therefore, the mean distance between the centres of the plates was 108 cm for men and almost 100 cm for women.

For each trial, the experimenter positioned the reference bar in one of the four predetermined orientations, and the test bar was oriented at either 70° or 120°. The experimenter placed the non-dominant hand of the participant above the reference bar and the dominant hand above the test bar. The task of the participant was to rotate the test bar in such a way that it felt parallel with respect to the reference bar, using both hands simultaneously and without switching hands while exploring and orienting the bars. There was no time limit set to perform the task and participants were not given feedback about their performance. After participants had set the test bar and were satisfied with the result, the experimenter recorded the orientation of the bar by reading the orientation indicated by the arrow-shaped end of the bar.

### Data analysis

The smallest deviation between the orientation of the test bar and the reference bar was the dependent variable. When deviations were counterclockwise to the reference bar, they were noted as negative values, with clockwise deviations being noted as positive values. This was reversed for the left-handed participant, with counter-clockwise deviations being noted as positive values and clockwise deviations as negative values. To study the effect of setting the test bar with the fingers, a repeated measurement ANOVA was performed with the following independent factors: condition (2: HPH and HPF), orientation (4: 0°, 45°, 90°, and 135°), and repetition (3 trials) as within-subject factors. A second analysis was performed including additionally gender (2: female and male participants) as between-subject factor. There was no violation of sphericity of the independent variables. Because of the small sample size, normality was tested using the Shapiro–Wilk test. The results showed that the deviations in both conditions were normally distributed in both genders. In addition, the deviations of the orientations in both conditions were normally distributed in both genders with the exception of the deviations for the 0° and 90° orientations for males in the HPF condition. This was due to the deviation of one male participant who had much larger deviations for these orientations in the HPF condition than the other male participants.

## Results

Analysis showed that there was no significant main effect of repetition [*F*(2, 36) = 1.94, *p* = 0.16], nor was there a significant interaction. Mean deviation for the first trial was 31.8°, for the second trial 33.7°, and for the third trial 34.5°. We, therefore, averaged over repetitions.

### Effect of condition and orientation

**Fig. 3:**
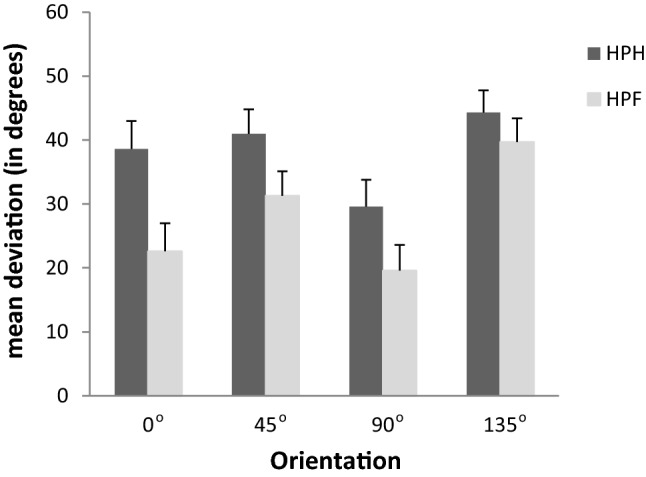
Mean deviations and standard errors for the different orientations in both conditions. *HPH* haptic parallelity task setting the test bar with the flat-stretched hand, *HPF* haptic parallelity task setting the test bar with the fingers

To study the effect of condition and orientation for all 20 participants, regardless of gender, a repeated measurement ANOVA was performed with condition and orientation as independent variables and deviation as dependent variable. This analysis showed significant main effects of condition [*F*(1, 19) = 9.49, *p* = 0.006] and orientation [*F*(3, 57) = 16.53, *p* < 0.001]. While a mean deviation of 38.4° was found in the condition, where the flat-stretched hand was used to set the test bar (HPH), a much smaller mean deviation of 28.3° was found in the condition, where the test bar was gripped with the fingers (HPF). Deviations for the different orientations were in line with earlier reported differences regarding cardinal and oblique orientations. The mean deviation for the cardinal orientation of 90° was smallest (24.6°) and largest for the oblique orientation of 135° (42.0°). The cardinal orientation of 0° resulted in a mean deviation of 30.6° with a mean deviation of 36.1° for the oblique orientation of 45°. Pairwise comparisons of the four orientations using a Bonferroni correction showed that the deviation of the 135° orientation was significantly different from the deviations of the other orientations (*p* = 0.002, *p* = 0.044, *p* < 0.001 for the 0°, 45°, and 90° orientations, respectively). In addition, the deviation for the 90° orientation was significantly different from the 45° orientation (*p* = 0.002). Although this pattern regarding deviations for the different orientations was the same in both conditions (see Fig. 3), a significant interaction of condition and orientation was found [*F*(3,57) = 4.55, *p* = 0.006]. Differences in deviations between both conditions were most pronounced for the 0° orientation (difference of 16°) and least for the orientation of 135° (difference of 4.6°).

### Effect of gender

**Fig. 4:**
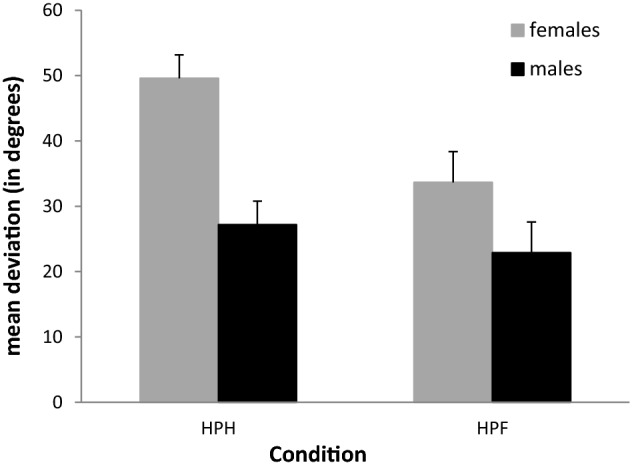
Mean deviations and standard errors for female and male participants in both conditions. *HPH* haptic parallelity task setting the test bar with the flat-stretched hand, *HPF* haptic parallelity task setting the test bar with the fingers

Including gender as a between-subject factor in the abovementioned repeated measurement ANOVA showed a main effect of gender [*F*(1,18) = 10.94, *p* = 0.004] as well as main effects of condition [*F*(1, 18) = 10.75, *p* = 0.004] and orientation [*F*(3, 54) = 16.15, *p* < 0.001]. While female participants had a mean deviation of 41.6°, a mean deviation of 25.0° was found for male participants. The interaction of condition and gender showed a trend [*F*(1,18) = 3.53, *p* = 0.07]. As can be seen in Fig. 4, differences in deviations between the genders were most pronounced when matching was done using the flat-stretched hand (HPH). In this condition, men had a mean deviation of 27.2° and women of 49.6°. Differences between the genders were smaller in the condition, where the test bar was set using the fingers (HPF). In this condition, male participants had a mean deviation of 22.9° and female participants of 33.7°. While male participants showed a small improvement of 4.3° when using the finger grip, a much larger improvement of 15.9° was observed for female participants.

**Fig. 5:**
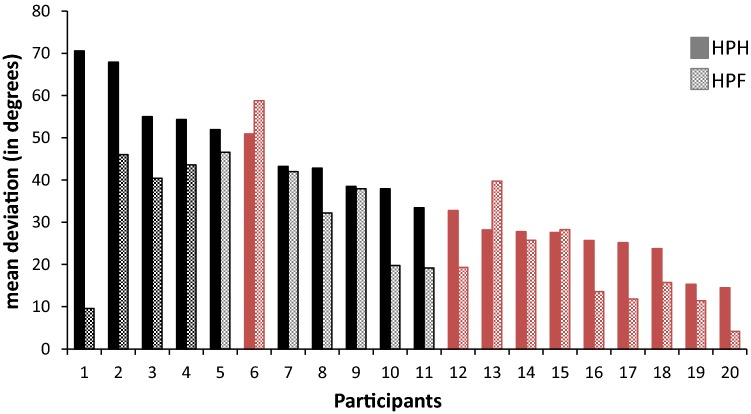
Mean deviations for both conditions for all participants. Filled bars represent *HPH* haptic parallelity task setting the test bar with the flat-stretched hand; dotted bars represent *HPF* haptic parallelity task setting the test bar with the fingers. Black bars display female participants, whereas red bars display male participants

Figure 5 shows the mean deviations for both conditions for all participants. As can be seen in this figure, all female participants had lower deviations in HPF compared to HPH, while some of the male participants showed the opposite with larger deviations in HPF. It can also be seen that not only participants with larger deviations (being mostly female participants) in the HPH condition benefitted most from using the fingers to match the orientation.

## Discussion

This study was conducted to investigate if reducing the influence of the hand-centered egocentric reference frame would decrease deviations in a pure haptic parallelity task. In this task, the orientation of a reference bar that was perceived with one hand had to be paralleled on a test bar that was manipulated with the other hand. To address this question, performance was assessed in a condition in which the test bar had to be matched using the flat-stretched hand and in a condition in which the test bar had to be rotated by gripping the bar with all fingers. It was hypothesized that in the latter condition, the bias of the hand-centered egocentric reference frame would be reduced, resulting in a decrease in deviations. The results support this hypothesis; deviations in the latter condition were significantly smaller than in the former.

We replicated previously reported results regarding haptic parallelity, with large and systematic deviations (e.g., Fernández-Díaz and Travieso 2011; Kaas and Van Mier 2006; Kappers 2003, 2018; Kappers et al. 2018; Van Mier 2013, 2016), as well as gender differences, with males outperforming females (Hermens et al. 2006; Kaas and Van Mier 2006; Kappers 2003, 2007; Van Mier 2013, 2016; Volcic et al. 2008; Zuidhoek et al. 2007). Although the intrinsic geometry of haptic space regarding the parallelity task is Euclidian (Cuijpers et al. 2003), these results clearly show that the perception of haptic space in this task is not. We also replicated earlier findings regarding orientation and obliqueness with cardinal orientations of 0° and 90° resulting in smaller deviations than the 45° and 135° oblique orientations (Hermens et al. 2006; Kaas and Van Mier 2006; Kappers 1999, 2002, 2003, 2004; Kappers and Viergever 2006; Van Mier 2013, 2016; Volcic et al. 2007).

Comparing the deviations in both conditions revealed that the deviations in the HPF condition were significantly smaller than in the HPH condition. Rotating the reference bar while using the fingers instead of the flat-stretched hand reduced the deviations by more than 26%. This is most likely due to a decrease in the egocentric bias of the hand. While both hands are aligned when the test and reference bar are felt and rotated by the flat-stretched hand, this is not the case when the reference bar has to be rotated using the fingers. In that case, it is most likely that an arm-centered reference frame is used. The current result is in line with earlier findings regarding the reduction of the bias of the hand in haptic parallel setting. Van Mier (2013) found a significant decrease in deviations when participants had to draw the orientation compared to haptically matching the orientation. Also in that condition, both hands were not aligned with an arm–shoulder reference frame most likely used when drawing the orientation. An important difference with the current study is that in the former visual information was available. While the test bar was blocked from their view, participants had full view of their drawing hand. One could argue that this stimulated the use of an allocentric reference frame rather than reducing the egocentric reference frame. Participants could use information from the sides of the plate and table and from doors and walls as an allocentric reference resulting in smaller deviations. However, Van Mier (2013) showed that just providing visual information by giving participants full view of their hand when matching the test bar, resulted in deviations that were significantly smaller than when haptically matching the reference bar, but still significantly larger than when drawing the orientation of the reference bar. While the visual information was the same in both conditions, the difference between the conditions was the reduction of the egocentric bias of the hand when drawing the orientation. The results of the current study corroborate the idea that the deviations in the regular haptic parallelity task are caused by the biasing influence of the hand-centered egocentric reference frame. Reducing this bias in a pure haptic condition resulted in smaller deviations. It is additionally possible that participants used some sort of allocentric cue in the HPF condition, by visualizing the orientation of the test bar or translating the orientation to, for instance, a clock time. Although participants did not mention that they used a specific strategy, they were not explicitly asked if they did. This might have increased the weighting factor of the allocentric reference frame.

While cardinal orientations resulted in smaller deviations than oblique orientations in both conditions, a significant interaction of condition and orientation was found. The orientation of 0° had considerably smaller deviations in the HPF condition than in the HPH condition than the other orientations. It is possible that this was due to biomechanical aspects. In this orientation, it is easier to rotate the flat-stretched hand in a more clockwise direction than when the bar is gripped with the fingers.

When two bars are horizontally far apart and each touched by one of the hands, the orientation of the hands will be different, as well as the position of the hands in relation to the body (Kappers and Viergever 2006). That brings up the question if the deviations are due to an influence of the body-centered reference frame or a hand-centered reference frame or both. The results of our study suggest that the deviations are mainly due to the hand-centered reference frame, because the hand at the reference bar is at the same position and rotation with respect to the body in both conditions. The orientation and rotation of the test hand in relation to the body are, however, not the same in both conditions, so there might still be an, although smaller, influence of the body-centered reference frame (Van Mier 2016).

It was additionally hypothesized that if gripping the reference bar with the fingers would result in smaller deviations, this effect would be most pronounced in female participants. It was found that deviations in the HPF condition were almost 16° smaller than in the HPH condition for women, while they were just a bit more than 4° smaller for men. In addition, all women showed smaller deviations in the HPF condition, while some men showed increases. However, the interaction of gender and condition was not significant, but only showed a trend. This result might even have been skewed by one of the female participants who showed an improvement of more than 60° when performing the task with the fingers. Excluding this participant resulted in an average improvement of 10.8° for the 9 female participants. Because both genders showed a similar improvement in the HPF condition, our second hypothesis was not confirmed. These results do not replicate earlier findings by Van Mier (2013, 2016) showing significantly larger decreases in deviations in female participants due to reducing or eliminating the egocentric bias of the hands. Having to draw the orientation of the test bar or to verbally name a coded orientation for the test bar, significantly reduced deviations for women in those studies. In addition, the mean difference in deviations between both conditions in the current study was smaller for male participants than reported in earlier studies by Van Mier (2013, 2016). Drawing the orientation or verbally reporting a coded orientation also resulted in much smaller deviations in male participants. These results might be due to the smaller sample size in the current study (only 10 participants in each gender group), compared to 30 participants in each gender group in the 2016 study of van Mier. Or, this might be attributable to differences in the setup of the studies. As stated before, in the earlier studies by Van Mier (2013, 2016), only the view of the reference bar was blocked, while participants could see their drawing hand or the protractor with the coded orientations. Differences in performance for women and men between those studies and the current study might be attributed to the additional use of allocentric cues due to visual information at the test side at the former, benefitting women more than men. Without visual information, the use of an allocentric reference frame is less stimulated.

Due to the fixed setup of the current study in which gripping and setting the test bar with all fingers was always performed after orienting the test bar with the flat-stretched hand, one could argue that the smaller deviations in the second condition are the result of a practice effect. However, if that would be the case, we would have expected a decrease in deviations as an effect of repetition. On the contrary, deviations did not decrease but actually increased over the three repetitions, although the effect was not significant. Hermens et al. (2006) did not find significant differences between participants who had heard about the systematic errors in the haptic parallelity task and participants who had not. In addition, Kappers and colleagues found that deviations did not significantly decrease when participants received haptic or visual training in the haptic parallelity task (Kappers et al. 2008). These findings show that the illusion of haptic parallelity is very strong. It is, therefore, very convincing that the decrease in deviations when gripping the test bar with the fingers is due to a reduction of the egocentric bias of the hands, rather than an effect of having performed the task before.

Taken together, the findings from the current study support the idea that the egocentric reference frame in the haptic parallelity task is mainly centered at the hand. Deviations from parallelity were much smaller when the bias of the hand was reduced and participants had to match the orientation by setting the bar using the fingers. This effect was found to be similar in female and male participants.
